# Topical Clonidine Accelerates Cutaneous Wound Healing in Diabetic Rats by Regulating the Expression of Related Cytokine Signaling

**DOI:** 10.3390/cimb46060339

**Published:** 2024-06-06

**Authors:** Thi-Chau-Loan Phan, Yi-Syuan Chiang, Ming-Jai Su, Yao-Jen Liang, Shiow-Jen Juang

**Affiliations:** 1 Graduate Institute of Applied Science and Engineering, Fu Jen Catholic University, New Taipei City 242, Taiwan; phanchauloan88@gmail.com (T.-C.-L.P.); 071558@mail.fju.edu.tw (Y.-J.L.); 2 Department of Life Science, Fu Jen Catholic University, New Taipei City 242, Taiwan; candy002712@gmail.com; 3 Graduate Institute of Pharmacology, National Taiwan University, Taipei City 106, Taiwan; mingja@ntu.edu.tw; 4 Department of Dermatology, Chi Mei Medical Center, Tainan City 710, Taiwan

**Keywords:** clonidine, wound healing, VEGF, SOCS3, JAK/STAT pathway

## Abstract

Based on the analgesic and anti-inflammatory effects of clonidine in previous studies, we hypothesized that clonidine could accelerate wound healing in rats by regulating the expression of related cytokines. In this study, the wound healing effect of clonidine was evaluated using an excision wound model in diabetic rats and a HaCaT cell model. The wounds were treated daily with topical clonidine. The results analyzed by ImageJ2 software show that the wounds of the rats that were treated with 15 ng/mL clonidine recovered faster, and the wound size was also significantly reduced compared to the control group. Western blot assays determined that clonidine induced an increase in the expression of vascular growth factors, namely, Ang-1, Ang-2, and VEGF. Moreover, clonidine demonstrated a rescuing effect on JAK2 within the JAK/STAT pathway by inhibiting SOCS3 expression, leading to decreased SOCS3 levels and increased expression of JAK2 and phospho-STAT3. Histopathological analysis revealed that clonidine promoted complete epithelial repair and minimized inflammation in skin tissue. Additionally, clonidine stimulated HaCaT cell proliferation in vitro and enhanced cellular energy levels in the presence of AGEs. In conclusion, clonidine promoted vascular growth and wound healing by stimulating the expression of cytokines that are beneficial for wound healing.

## 1. Introduction

Clonidine is an alpha-2 receptor agonist that downregulates the sympathetic nervous system. Clonidine is used to treat high blood pressure, attention deficit hyperactivity disorder, drug withdrawal, and certain pain conditions. According to the study of Figueroa et al. in 2001, clonidine activates the endothelial alpha(2)-adrenoceptor receptor, thereby releasing nitric oxide (NO) in the mesenteric artery of Sprague Dawley (SD) rats [1]. NO plays a key role in relaxing blood vessels, and reducing oxidative stress, and it also represents a potential wound therapeutic agent due to its ability to regulate inflammation and bacterial infections [2,3]. Clonidine was introduced into the clinical routine in 1966; since then, clonidine has been widely researched and used for its many preeminent effects and safety [4]. Clonidine has been studied extensively for its pain-relieving properties [5,6,7,8], and there has been limited research specifically examining the effects of clonidine on wound healing [9]. Based on this background, we conducted experiments to evaluate the effect of clonidine on wound healing in a diabetic rat model. This is a potential study on this old drug, and a new and very different topical development direction of clonidine.

Type 2 diabetes is on the rise within the community due to the prevalence of unhealthy dietary patterns and lifestyle choices [10]. For individuals with long-standing diabetes, injury is inevitable, especially for the elderly and those who are bedridden. People who have diabetes are also immunocompromised; therefore, chronic wounds often occur in patients with diabetes mellitus due to the impairment of wound healing [11]. Traditionally, wound healing has been divided into three distinct phases: inflammation, proliferation, and remodeling. The initial inflammatory phase involves vasoconstriction, platelet aggregation, and cellular influx, aiming to stop bleeding, clear debris, and prevent infection. This is followed by a proliferative phase characterized by granulation tissue formation, epithelialization, and neovascularization, lasting several weeks. Finally, the maturation and remodeling phase ensue, achieving maximum wound strength [12]. Patients with diabetes experience impaired wound healing due to a complex interplay of factors involving the structure, biochemistry, cells, and microbes. Hyperglycemia and the resulting inflammation disrupt the immune system, damage blood vessels, cause nerve damage, and lead to cellular aging. This inhibits the transition from the inflammatory stage, disrupts the microbiome, hinders the formation of the extracellular matrix, and creates imbalances in growth factors and cytokines. Moreover, it limits the regeneration of epithelial tissue and alters the movement and growth of fibroblasts [13]. Chronic wounds increase the cost of treatment, cause pain and stress for the patient, and become a burden on the family, the medical system and the whole society. One of the most important factors triggering the complications of chronic diabetes is the excess of Advanced Glycation End products (AGEs). The Maillard hypothesis mentioned that diabetic vascular complications are caused by the accelerated accumulation of AGEs in long-lived tissue [14]. When AGEs accumulate in the basement membrane, they will reduce antibacterial ability, hinder the oxidation process, adversely affect collagen formation, cause tissue hardening and slow the wound healing process [15]. In this study, we evaluated the wound healing efficacy of clonidine using a diabetic rat model and cell model supplemented with AGEs.

Wound healing is not a simple phenomenon and involves a complex interplay between numerous cell types, cytokines, mediators, and the vascular system [16]. In this study, we investigated the increase or decrease in proteins involved in the inflammatory response and wound healing, including angiopoietin-1 (Ang-1), angiopoietin-2 (Ang-2), vascular endothelial growth factor (VEGF), suppressor of cytokine signaling 3 (SOCS3), Janus kinase 2 (JAK2), signal transducer and activator of transcription 3 (STAT3), and cluster of differentiation 68 (CD-68). In many previous studies, to mimic diabetic conditions in in vitro experiments, mammalian cells were usually cultured in medium supplemented with AGEs [17,18,19]. For this study, we also examined the effect of clonidine on the proliferation and viability of cultured HaCaT cells in the presence of AGEs.

## 2. Materials and Methods

### 2.1. Materials and Methods for the In Vitro Experiment

Cell line: The human epidermal keratinocyte (HaCaT) cell line was purchased from American Type Culture Collection, Manassas, VA, USA with database name: primary epidermal keratinocytes; normal, human, adult; accession numbers: PCS-200-011™. HaCaT cells were cultivated in DMEM supplemented with 10% fetal bovine serum and 1% penicillin‒streptomycin (Gibco™, Waltham, MA, USA) and kept in an incubator at 37 °C in a humidified atmosphere with 5% CO_2_. This standardized cultivation regimen ensures optimal conditions for the proliferation and maintenance of HaCaT cells, preserving their characteristic properties throughout the experimental procedures.

Cell proliferation assay: HaCaT cells were seeded in 96-well plates at a density of 1 × 10^5^ cells/cm^2^. The cells were then treated with 5 ng/mL, 15 ng/mL and 25 ng/mL clonidine within 48 h, the experiment was conducted with 3 repetitions. The number of living cells was determined by direct staining and counting under a microscope.

Cell viability assay: HaCaT cells were seeded in 96-well plates at a density of 1 × 10^5^ cells/cm^2^ and treated with 5 ng/mL, 15 ng/mL and 25 ng/mL clonidine within 30 min. After that, 20 μL of Advanced Glycation End products (AGEs, 100 μL AGEs/1 mL cell culture) including 25 mM glyceraldehyde and 1 mM diethylene triaminepenta acetic acid was added to each 200 μL cell culture, the experiment was conducted with 3 repetitions. The total amount of nicotinamide adenine dinucleotide (NADH) and nicotinamide adenine nucleotide phosphate (NADPH) produced by the cells in each well was determined by the Cell Counting Kit-8 (MedChem Express, Monmouth Junction, NJ, USA) with exposure time 2 h.

### 2.2. Materials and Methods for the In Vivo Experiment

Animals: Adult male SD rats weighing 200 to 250 g were purchased from Lesco Biotechnology Company, Taipei, Taiwan. All experimental procedures were approved by the Fu Jen Laboratory Animal Center, Fu Jen Catholic University (IACUC No. FJU A10526). To help mice adapt to their new environment, one week prior to the beginning of the experiment, the mice will be kept in cages at 25 °C at room temperature and 45% humidity with a 12:12 light–dark cycle, and food and fresh water was provided ad libitum. Rats were divided into 3 groups: 1 control group, 1 group treated with clonidine at a concentration of 15 ng/mL, and 1 group treated with clonidine at a concentration of 25 ng/mL.

Surgical procedure: During the experiments, SD rats were fed a high-fat diet (92% feed powder, 7% lard, 1% cholesterol). To induce diabetes in rats, a dose of streptozotocin at a final concentration of 4 mg/mL (Sigma Inc., St. Louis, MO, USA) was injected into the peritoneum at a dose of 70 mg/kg. One week after the injection, the rats with blood glucose levels above 250 mg/dL were considered to have diabetes. After anesthesia, the backs of the rats were shaved and antisepsis was performed with 70% alcohol. All rats had skin square excision wounds of 1 cm^2^.

Drug treatment: A 10 µg/mL stock solution of clonidine was prepared by dissolving clonidine in sterilized distilled water (ddH_2_O). The topical ointment containing clonidine was formulated as follows: dissolve either 150 µL or 250 µL of 10 µg/mL stock solution in 93 mL of ddH_2_O, then add 2 mL of Creagel emulsifier (First Chemical, Taipei, Taiwan), and finally, 5 mL of glycerol (Sigma Inc., MO, USA). An ointment containing clonidine (approximately 0.5 mL) was applied to the 1 cm^2^ wounds daily. A similar formula ointment was applied to the control group, excluding clonidine. The wounds were observed and captured under a DinoCapture photomicroscope on days 1 (pre-treatment) and 3, 5, 7, 9, 11, and 13 post-treatment. This systematic approach ensures a comprehensive evaluation of the wound healing process, facilitating the discernment of any discernible effects attributed to clonidine administration.

Euthanasia method using Carbon dioxide: Exposing animals to CO_2_ without relocating them from their home cage is a swift and humane euthanasia method. This approach minimizes stress since the animals are not subjected to handling or the disturbance of being transferred to a new environment.

Place 2 rats into the CO_2_ chamber for euthanasia. The procedure involved administering 100% CO_2_ into the chamber, adhering to AVMA guidelines [20]. CO_2_ was introduced at a rate of 50% of the chamber volume per minute for a total of 5 min. Confirmed death following exposure to CO_2_ was determined through a meticulous evaluation of the animal, focusing on unequivocal indicators of death, such as cardiac arrest or fixed, dilated pupils. In the event of any uncertainties and to ensure complete euthanasia, a secondary method was employed by opening the chest cavity. On the 13th day post-treatment, the wound and surrounding intact skin, approximately 1 cm^2^ were harvested. Subsequently, the collected samples underwent fixation in formaldehyde 4%, ensuring optimal preservation of tissue integrity, followed by cryopreservation for further detailed analysis.

Western blot analysis: A protein extraction solution supplied by iNtRON Biotechnology (Serdang, Malaysia) was used to extract total protein from collected tissue samples. The BCA method was used to determine the total protein concentration. An equivalent of 30 μg of total protein was separated by 12% SDS-PAGE and then transferred to a PVDF membrane (Merck Millipore, Burlington, MA, USA). After blocking with 5% non-fat milk for 1 h, the membranes were incubated overnight at 4 °C with the following primary antibodies (Santa Cruz Biotechnology, Santa Cruz, CA, USA) mouse-anti-: VEGF-C1 (1:500), Ang-1 (1:200), Ang-2 (1:500), JAK2 (1:200), total STAT3 (1:500), phospho-STAT3 (1:500), CD68 (1:200), SOCS3 (1:200), and β-actin (1:500). The membranes were washed with PBST five times and incubated with goat anti-mouse IgG (Heavy Chain) secondary antibody HRP (Thermo Fisher Scientific, Waltham, MA, USA, 1:10,000) for 1 h at room temperature. The experiment was conducted with 6 repetitions. Signals were visualized using the UVP ChemiDoc-It machine (UVP, Upland, CA, USA), and target protein and β-actin bands were analyzed using ImageJ2 software (LOCI, University of Wisconsin).

Histopathological analysis: all samples were fixed in formaldehyde 4% followed by embedding in paraffin, sectioned at 3–5 μm thickness, and stained with hematoxylin and eosin (H&E). Images were acquired using the 3D Histech Pannoramic 250 Flash III scanner (3DHISTECH Ltd., Budapest, Hungary) at 50× magnification. The recovery level of the wound is evaluated according to the following criteria (Table 1):

### 2.3. Data Analysis

The Statistical Package for the Social Sciences (SPSS) version 22 software was used to analyze the collected data. All data will be presented as the mean ± standard error of the mean (SEM) of *n* observations, where *n* represents the number of repetitions of cells per group or the number of animals per group. Statistically significant differences between the control group and the treated groups as well as each treated group with others were tested using one-way ANOVA followed by the Scheffe multiple comparison test, and *p* < 0.05 was considered to be statistically significant.

## 3. Results

### 3.1. Clonidine Stimulated HaCaT Cell Proliferation

**Figure 1:**
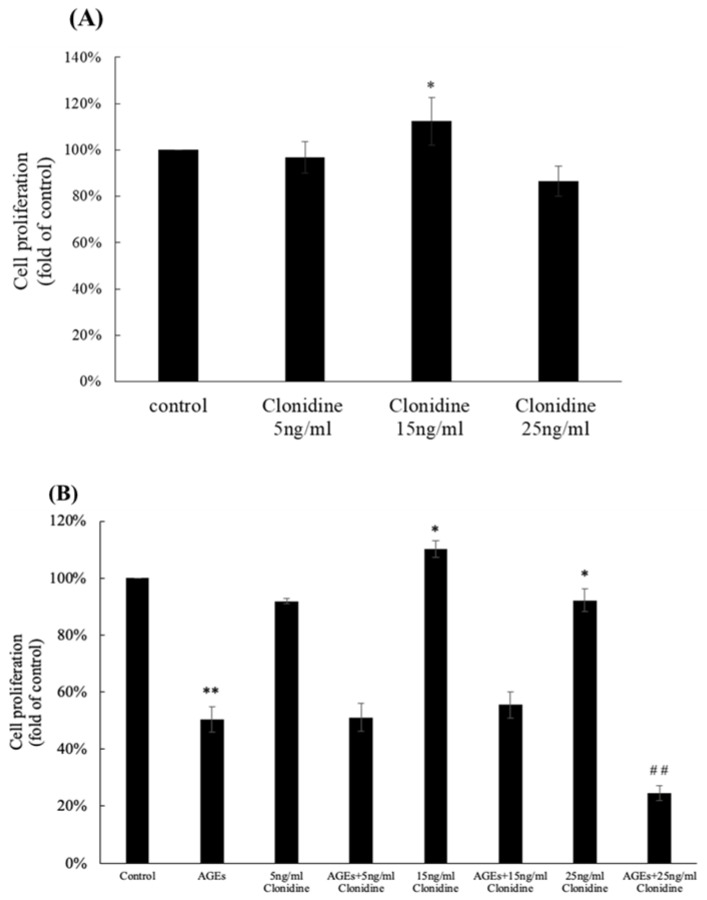
The effects of clonidine on HaCaT cell proliferation. (**A**) Clonidine stimulated HaCaT cell proliferation. (**B**) Clonidine did not stimulate the proliferation of HaCaT cells in the presence of AGEs; * *p* < 0.05, ** *p* < 0.01 vs. the control group; ^##^ *p* < 0.01 vs. the AGE group.

In assessing the impact of clonidine on HaCaT cell proliferation, Dulbecco’s Modified Eagle Medium (DMEM) was supplemented with clonidine at varying concentrations: 5 ng/mL, 15 ng/mL, and 25 ng/mL. The quantification of viable cells transpired through direct staining and microscopic counting. As depicted in Figure 1A, the medium fortified with 15 ng/mL clonidine conspicuously augmented cell numbers. Intriguingly, at a heightened concentration of 25 ng/mL, clonidine failed to elicit a positive effect; instead, it significantly impeded the proliferation of HaCaT cells.

To emulate diabetic conditions within cultured cells, Advanced Glycation End products (AGEs) were introduced to DMEM. Subsequent treatment involved clonidine concentrations of 5 ng/mL, 15 ng/mL, and 25 ng/mL. Remarkably, AGEs themselves demonstrated an inhibitory effect on HaCaT cell proliferation. Intriguingly, under the influence of AGEs, clonidine did not exert its effect on cell proliferation, as illustrated in Figure 1B. This result indicates that clonidine exhibits a proliferative effect on HaCaT cells at a concentration of 15 ng/mL in the absence of AGEs.

### 3.2. Clonidine Enhanced the Viability of HaCaT Cells In Vitro in the Presence of AGEs

**Figure 2:**
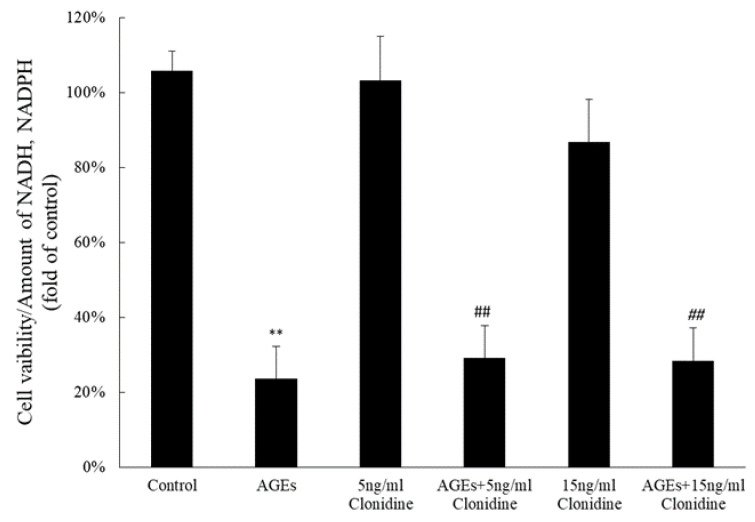
Effect of Clonidine on cell viability in the presence or absence of AGEs. Clonidine enhanced the synthesis of NADH and NADPH in HaCaT cells in vitro in the presence of AGEs; ** *p* < 0.01 vs. the control group; ^##^ *p* < 0.01 vs. the AGE group.

Within the medium supplemented with AGEs, HaCaT cells responded robustly to clonidine, exhibiting a notable augmentation in the synthesis of both NADH and NADPH. This effect was particularly pronounced at concentrations of 5 ng/mL and 15 mg/mL, as delineated in Figure 2. The observed increase in these crucial cellular cofactors signifies the capacity of clonidine to enhance the cellular vitality of HaCaT cells even in the presence of AGEs, thereby showcasing its potential as a modulator of cellular resilience and metabolic processes.

### 3.3. Clonidine Accelerated Wound Healing in Diabetic Rats

In this study, diabetic rats with square excision wounds on their skin were subjected to daily treatment with varying concentrations of topical clonidine, specifically 15 ng/mL and 25 ng/mL. The progression of wound healing was meticulously monitored by capturing images at three-day intervals and subsequently analyzing them using the advanced ImageJ software developed by LOCI at the University of Wisconsin.

**Figure 3:**
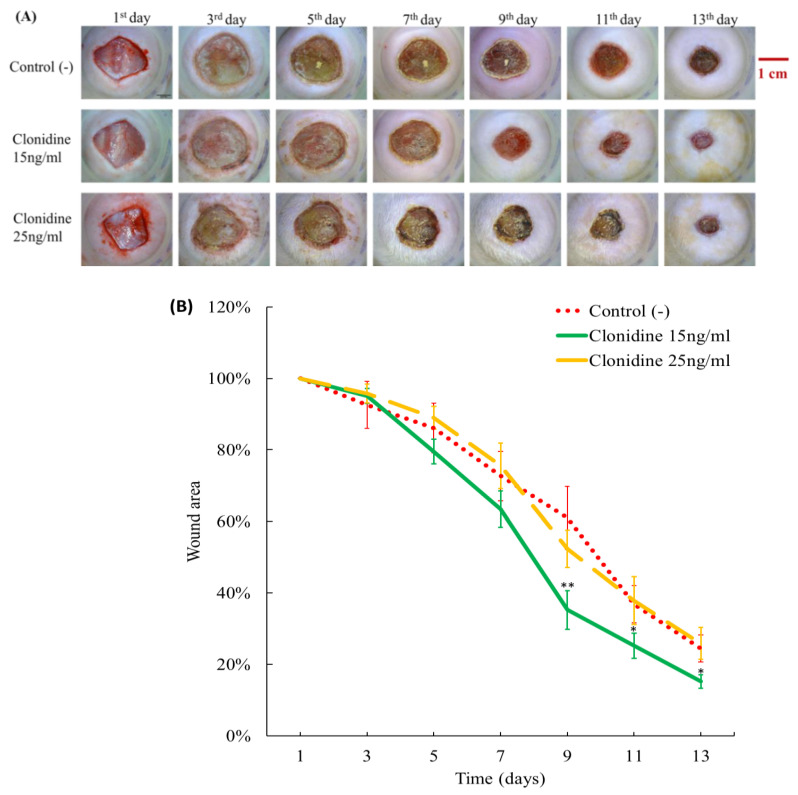
The differences in wound healing in rats in three groups: control (-), treated with clonidine at concentrations of 15 ng/mL and 25 ng/mL. (**A**) The wounds of the rats treated with 15 ng/mL clonidine healed faster compared to those in the untreated group. (**B**) The wound images were analyzed using ImageJ software. The difference between the group that was treated with 15 ng/mL clonidine and the control group could be clearly recognized at days 9, 11 and 13; * *p* < 0.05, ** *p* < 0.01 vs. control.

At a clonidine concentration of 15 ng/mL, substantial enhancements were consistently noted throughout the entire observation period. The wounds exhibited a persistently drier surface, maintaining a smaller size in comparison to the control group, as visually illustrated in Figure 3A. Notably, a substantial reduction in wound size became apparent starting from the 9th day of treatment, displaying round, clean, and dry characteristics, with the surrounding skin appearing ruddy, even, and bright. This visual transformation underscores the significant positive impact of clonidine on wound healing dynamics.

Quantitative analysis of the images using ImageJ software, as illustrated in Figure 3B, provides a comprehensive visualization of the positive impact of clonidine on wound healing in diabetic rats. By the 13th day, the wound size in the clonidine-treated group was approximately halved compared to the control group, underscoring the robust efficacy of clonidine in promoting accelerated wound recovery in diabetic conditions. These findings affirm the therapeutic potential of clonidine as a valuable agent for enhancing wound healing outcomes in diabetic rats.

### 3.4. Clonidine Regulated the Expression of Cytokines Involved in the Inflammatory Response and Wound Healing

**Figure 4:**
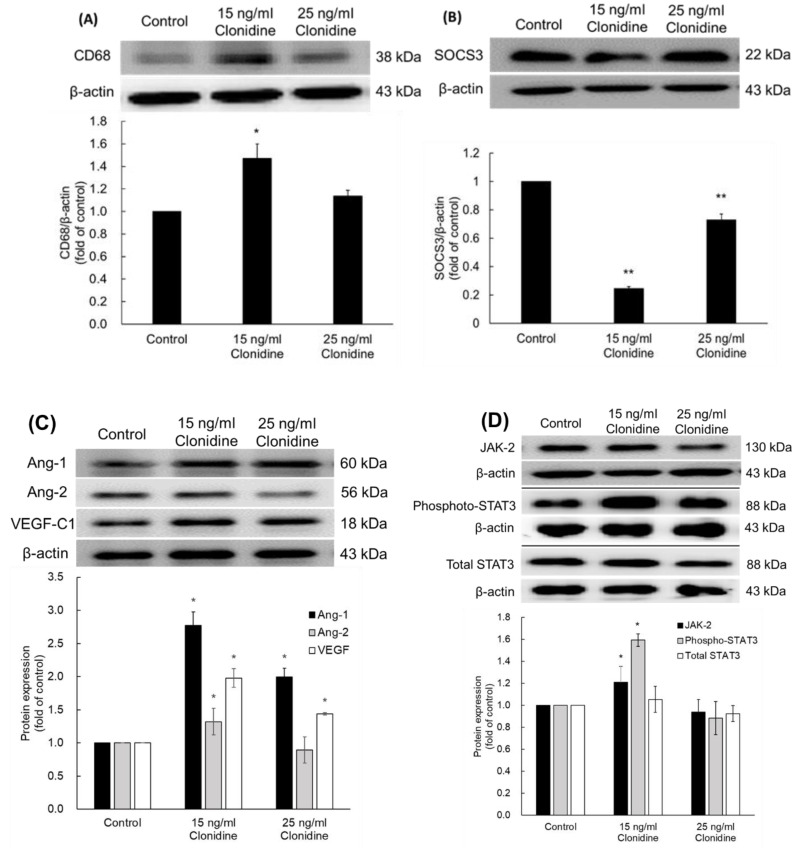
The expression of proteins involved in inflammatory response and wound healing under clonidine treatment at the concentrations of 15 ng/mL and 25 ng/mL, compared to the untreated group. (**A**) Under the effect of clonidine, the expression of CD68 was increased on macrophages. (**B**) Clonidine induced a decrease in the expression of SOCS3. (**C**) The 15 ng/mL clonidine treatment led to an increase in the expression of vascular growth factors, including Ang-1. Ang-2 and VEGF. (**D**) The expression of JAK2 and STAT3 was found to increase in response to 15 ng/mL clonidine treatment; * *p* < 0.05, ** *p* < 0.01 vs. the control group.

Considering the favorable outcomes demonstrating efficacy of clonidine in promoting wound healing, a comprehensive exploration into the molecular mechanisms governing this phenomenon was undertaken through Western Blot assays. Clonidine, administered at a concentration of 15 ng/mL, exhibited a remarkable induction in the expression of CD68 on macrophages, signifying an enhanced macrophage activation. Intriguingly, when the concentration was elevated to 25 ng/mL, no substantial change in CD68 levels was discerned, as depicted in Figure 4A.

Detailed scrutiny of Figure 4B,D unraveled intricate molecular dynamics within the 15 ng/mL clonidine-treated group. Herein, the abundance of suppressor of cytokine signaling 3 (SOCS3) witnessed a profound fourfold reduction, coupled with a significant upregulation in Janus kinase 2 (JAK2) and phosphorylated signal transducer and activator of transcription 3 (phospho-STAT3). Notably, the levels of total STAT3 remained unaltered. In contrast, the 25 ng/mL clonidine-treated group exhibited a reduction in SOCS3 levels, while JAK2 and STAT3 showed no significant alteration.

Figure 4C delves into the impact of clonidine on vascular growth factors. In the 15 ng/mL clonidine-treated group, a substantial elevation was observed in angiopoietin-1 (Ang-1), angiopoietin-2 (Ang-2), and vascular endothelial growth factor (VEGF) levels, indicative of a robust pro-angiogenic effect. Intriguingly, in the 25 ng/mL clonidine-treated group, only Ang-1 and VEGF exhibited significant increases, underscoring nuanced concentration-dependent effects.

Collectively, these intricate datasets provide compelling evidence that clonidine orchestrates the regulation of key proteins integral to both the inflammatory response and wound healing. The nuanced dose-dependent alterations in CD68, SOCS3, JAK2, phospho-STAT3, and vascular growth factors underscore the multifaceted and targeted role of clonidine in establishing a conducive microenvironment for expeditious wound recovery.

### 3.5. Clonidine Promoted Complete Epithelial Repair and Minimal Inflammation on Skin Tissue

**Figure 5:**
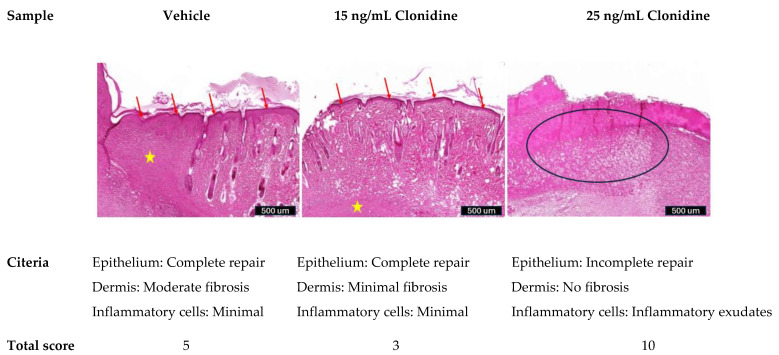
H&E staining method compares wound recovery under clonidine treatment at the concentrations of 15 ng/mL and 25 ng/mL, compared to the untreated group. Clonidine promoted complete epithelial repair and minimal Inflammation on skin tissue at the concentration of 15 ng/mL. The red arrow indicates epithelial repair. The yellow star represents the dense fibrosis parts shown in the H&E stain. Finally, the black circle highlights the inflammatory exudates in the group treated with 25 ng/mL of clonidine.

Figure 5 illustrates skin tissue exhibiting injury and inflammation changes. To assess repair status semi-quantitatively, three sections of the skin tissue were evaluated. The vehicle group displayed complete epithelial repair but moderate dermal fibrosis, indicating inadequate tissue repair with residual fibrosis. The group treated with 15 ng/mL clonidine exhibited complete epithelial repair, minimal dermal fibrosis, and minimal inflammatory cell infiltration, indicative of effective tissue repair. However, at a concentration of 25 ng/mL clonidine, the wound exhibited incomplete epithelial repair, with only inflammatory exudates present and no dermal fibrosis.

## 4. Discussion

The in vitro experiments showed the impact of clonidine on HaCaT cell proliferation, revealing distinct dose-dependent effects. At a concentration of 15 ng/mL, clonidine exhibited a notable promotion of HaCaT cell proliferation. Intriguingly, at a higher concentration of 25 ng/mL, a contrasting effect was observed, wherein clonidine seemed to inhibit cell proliferation. This bidirectional response suggests a nuanced relationship between clonidine concentration and HaCaT cell behavior. The inhibitory effect at higher concentrations may be attributed to the potential interference with crucial proteins in cellular metabolic and divisional pathways. This intricate modulation of cellular responses underscores the need for a meticulous examination of clonidine concentrations to optimize its therapeutic benefits without compromising cellular functions. Further investigations into the specific proteins affected could provide valuable insights into the underlying mechanisms governing clonidine’s dose-dependent effects on HaCaT cell proliferation. Interestingly, in the presence of Advanced Glycation End products (AGEs), clonidine did not induce an increase in cell proliferation; however, it markedly augmented the synthesis of NADH and NADPH in HaCaT cells. It is possible that AGEs induce cellular stress; then, under the influence of clonidine, the stressed cell has prioritized energy production and the recovery of stressed cells over proliferation. NADH and NADPH play crucial roles in cellular metabolism. NADH is the reduced form of NAD^+^ and is involved in electron transfer processes during cellular respiration, serving as the main carrier of electrons that convert food into ATP, the basic energy source of the cell. NADPH is the reduced form of NADP^+^ and is important in redox and biosynthetic processes in the cell. It is used in the synthesis of fatty acids, cholesterol, steroids, vitamins, nucleic acids, and other compounds. NADPH also plays a critical role in protecting cells from damage and maintaining the stability of cell membranes [21]. In this study, clonidine emerged as a potent modulator of cellular metabolism, specifically as an inducer of NADH and NADPH synthesis, thereby contributing to cellular metabolism and the recuperation of stressed cells. This intricate interplay between clonidine, AGEs, and cellular metabolic processes unravels the nuanced mechanisms through which clonidine interfaces with cellular dynamics, presenting opportunities for targeted therapeutic interventions aimed at optimizing cellular resilience and metabolic homeostasis.

Many previous studies have demonstrated that clonidine plays a role in stimulating the secretion of growth hormone (GH) [22,23,24,25,26]. This orchestrated endocrine modulation, initiated by clonidine, precipitates a cascade of intricate physiological events that profoundly influence wound healing. Following GH induction, the reparative cascade unfolds with GH playing a central role in augmenting collagen synthesis, a linchpin in the preservation of tissue integrity and strength. The therapeutic impact of GH extends to finely regulating essential growth factors within the wound microenvironment. Noteworthy among these factors are vascular endothelial growth factor (VEGF), Fibroblast Growth Factor (FGF), and Stromal cell-Derived Factor-1 (SDF-1), collectively orchestrating tissue regeneration and angiogenesis. GH, functioning as a regulatory maestro, elevates the expression and availability of these growth factors, thereby fostering an environment optimally conducive to accelerating wound healing [27]. In another study, independent of GH, clonidine demonstrated an increase in VEGF and VEGF receptor expression, along with a concurrent reduction in lung inflammation, ultimately culminating in the amelioration of lung tissue repair [9]. In this study, at a concentration of 15 ng/mL, clonidine showed a positive role in wound treatment. Remarkably, by the 13th day, the wound size was only approximately half that of the no-treatment group. In a study by Tyler J. Loftus et al., using an SD rat model of lung injury with varying degrees of lung contusion (LC), lung contusion/hemorrhagic shock (LCHS), or lung contusion/hemorrhagic shock/daily restraint stress (LCHS/CS), clonidine was found to enhance VEGF expression by 43% in cases of LCHS and 46% in cases of LCHS/CS. Additionally, clonidine increased VEGFR-1 and R-2 expression by 203% and 47%, respectively, following LCHS/CS [9]. In our study, Western blot analysis revealed significant increases in all three types of vascular growth factors including Ang-1, Ang-2, and VEGF. Specifically, at a clonidine concentration of 15 ng/mL, Ang1 showed a remarkable increase of 178%, Ang-2 increased by 32%, and VEGF by 98%. At a higher concentration, 25 ng/mL of clonidine, Ang1 exhibited a 100% increase, while VEGF increased by 44%. This substantial enhancement in growth factor levels provides a explanation for the accelerated wound healing observed in rats treated with 15 ng/mL clonidine, surpassing the recovery rate observed in the control group.

An additional consequence of the growth hormone (GH) induction mediated by clonidine is the activation of the JAK2 receptor and the ensuing initiation of the JAK/STAT signaling pathway [28]. This signaling pathway, widely expressed intracellularly, assumes a pivotal role in fundamental biological processes such as proliferation, differentiation, and immune regulation. Of particular significance is its intricate involvement in wound healing, where the JAK/STAT pathway plays a multifaceted role. It orchestrates complex cellular responses, modulating inflammation, promoting tissue repair, and regulating various aspects of the wound healing cascade, thereby exerting a substantial influence on the overall regenerative processes during tissue recovery [29]. In a study on *Periplaneta americana* extract (PAE), an extract that plays a crucial role in skin wound healing, the results showed that the HaCaT cells that were treated with PAE had significantly higher JAK2 expression, and the amount of phospho-JAK2 was also increased, while STAT3 and phospho-STAT3 remained unchanged [30]. The results of this study indicated a significant increase in both JAK2 and phospho-STAT3, which can be explained by differences in the research models. An in vivo model would be more complex and have multiple reactions than an in vitro model, leading to an increase in various cytokines. In general, these increases are beneficial to the wound healing process. Involved in the JAK/STAT pathway, SOCS3, plays a crucial role in modulating cytokine or hormone signaling, typically acting as a safeguard, but in certain instances, exacerbating diverse diseases. Its primary function stems from its ability to bind to both the JAK kinase and the cytokine receptor, leading to the suppression of STAT3 activation [31]. A clinical study of Yi Feng et al. revealed markedly elevated levels of SOCS3 in non-healing chronic wounds compared to healing/healed chronic wounds at the transcript level [32]. Therefore, intervening to curtail prolonged overexpression of SOCS3 holds promise for enhancing chronic wound healing and tissue regeneration. The results of this study demonstrated a notable decrease in SOCS3 levels in rat tissue under the influence of clonidine. This finding suggests that clonidine exerts its influence by mitigating the inhibitory effects of SOCS3 on the JAK/STAT pathway, thereby facilitating the rescue of JAK2 functionality within this signaling pathway.

Macrophages are key innate immune cells that play a significant role during wound healing, including host defense, the promotion and resolution of inflammation, the removal of apoptotic cells, and the support of cell proliferation and tissue restoration following injury [33]. As a quintessential macrophage biomarker, CD-68 provides insights into macrophage activation levels. The investigation indirectly probed macrophage activation by assessing CD68 expression. The results in Figure 4A show that 15 ng/mL topical clonidine induced an increase in the expression of CD68 on macrophages. However, when macrophages are inappropriately activated, they backfire, such as in fibrosis or chronic nonhealing wounds [34]. Thus, the number of macrophages activated by clonidine also needs to be investigated further.

As the concentration of clonidine escalates to 25 ng/mL, a discernible diminution in its positive efficacy becomes apparent. This observation aligns seamlessly with the outcomes of the Western blot analysis, where heightened clonidine concentrations failed to elicit an upregulation in cytokines known for their beneficial roles in wound healing. This intriguing phenomenon is suggestive of a potential threshold beyond which the positive effects of clonidine may be compromised. Plausible explanations for this diminishing effectiveness could revolve around the possibility that elevated clonidine concentrations may instigate adverse side effects. It is conceivable that clonidine, at higher concentrations, may interfere with the activities of pivotal proteins crucial for wound healing, or alternatively, reversible inhibition might occur due to an excessive drug concentration threshold. Delving into the intricate interplay between clonidine concentrations and the molecular pathways involved in wound healing may uncover subtle regulatory nuances, paving the way for more targeted and effective therapeutic interventions in the future.

**Figure 6:**
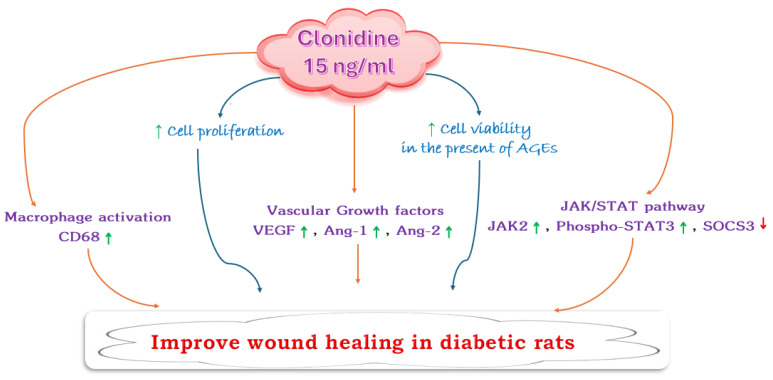
Summary of the effects of clonidine on wound healing in diabetic rats through in vitro and in vivo experiments, the red down arrow represents a decrease or inhibition, the green up arrow represents an increase or stimulation.

Histopathological analysis serves as a crucial tool for assessing tissue responses to therapeutic interventions and guiding the development of novel pharmaceutical agents. The histopathological analysis of this study (Figure 5) revealed distinct differences between the vehicle group and the groups treated with varying concentrations of clonidine. In the vehicle group, despite observing complete epithelial repair, the presence of moderate dermal fibrosis suggests inadequate tissue repair, with residual fibrosis remaining. This finding underscores the limitations of natural wound healing processes and highlights the need for interventions to enhance tissue regeneration. Conversely, treatment with 15 ng/mL clonidine resulted in remarkable improvements in tissue repair. The observed complete epithelial repair, along with mild dermal fibrosis and minimal inflammatory cell infiltration, signifies effective wound healing. These results suggest that clonidine at this concentration promotes a favorable environment for tissue regeneration, facilitating efficient epithelial repair while minimizing inflammation and fibrosis. However, it is noteworthy that the efficacy of clonidine appears to be concentration dependent. At a higher concentration of 25 ng/mL, incomplete epithelial repair was observed, accompanied by the presence of inflammatory exudates and absence of dermal fibrosis. This outcome raises questions regarding the optimal dosage of clonidine for promoting wound healing, as higher concentrations may potentially impede the repair process. In summary, the results from both in vivo and in vitro studies demonstrate that clonidine, at a concentration of 15 ng/mL, effectively promotes wound healing. This effect is achieved through stimulating cell proliferation and viability, regulating the expression of proteins involved in the inflammatory response and wound healing, and facilitating complete epithelial repair with minimal inflammation in skin tissue (Figure 6).

It is essential to conduct a comparative study to assess the wound healing effects of topical clonidine compared to commonly used dressings. Additionally, in our next study, we will explore incorporating clonidine as an adjunct component into various types of dressings. This approach aims to provide valuable insights into both the efficacy and practical application of clonidine. The optimal concentration of clonidine to mitigate unwanted side effects remains unclear, serving as a limitation of this study. This aspect will be addressed in future research endeavors.

## 5. Conclusions

This study demonstrates that clonidine positively influences wound healing in diabetic rats by regulating inflammatory responses and proteins related to wound healing. It underscores the complexity of the wound healing process and the necessity for optimal levels of various factors to minimize scarring, thereby advancing the field of regenerative medicine by revealing the therapeutic potential of clonidine. These findings pave the way for developing more refined therapeutic strategies for wound healing and tissue regeneration. While previous research has predominantly focused on clonidine’s analgesic properties, its impact on wound healing has received limited attention. This study highlights the need for further investigation into the dual role of clonidine in both analgesia and wound recovery. Additionally, future studies will explore the potential adverse effects of high clonidine concentrations on the wound healing process to ensure safe and effective therapeutic applications.

## Figures and Tables

**Table 1:** Criteria for evaluating the extent of wound healing.

Parameter	Epithelium	Dermis	Inflammation
Score 1	Complete	Minimal	Minimal
Score 2	Incomplete	Mild	Mild
Score 3	A few squamous cells	Moderate	Moderate
Score 4	No squamous cells	No fibrosis	Inflammatory exudates
The lower the score of the sample, the more complete the repair of the skin tissue.			

## Data Availability

The data supporting the findings of this study are available upon request. Please contact the corresponding author for access to the data.
